# Effect of Temperature on the Germination of Five Coastal Provenances of *Nothofagus* *glauca* (Phil.) Krasser, the Most Representative Species of the Mediterranean Forests of South America

**DOI:** 10.3390/plants11030297

**Published:** 2022-01-24

**Authors:** Rómulo E. Santelices-Moya, Marta González Ortega, Manuel Acevedo Tapia, Eduardo Cartes Rodríguez, Antonio M. Cabrera-Ariza

**Affiliations:** 1 Centro de Desarrollo del Secano Interior, Facultad de Ciencias Agrarias y Forestales, Universidad Católica del Maule, Talca 3466706, Chile; rsanteli@ucm.cl; 2 Centro Tecnólogico de la Planta Forestal, Instituto Forestal Sede Biobío, Camino a Coronel Km 7.5, San Pedro de la Paz 4130000, Chile; mgonzale@infor.cl (M.G.O.); macevedo@infor.cl (M.A.T.); ecartes@infor.cl (E.C.R.); 3 Centro de Investigación y Estudios Avanzados del Maule, Vicerrectoría de Investigación y Postgrado, Universidad Católica del Maule, Talca 3466706, Chile

**Keywords:** hualo, Mediterranean plants, seeds

## Abstract

Temperature is one of the most important abiotic factors affecting seed germination, and it is strongly influenced by local site conditions. Seeds of *Nothofagus* *glauca*, an endemic and vulnerable species of the Mediterranean region of Chile and the most representative of the Mediterranean forests of South America, were collected. In this study, we evaluated the effect of temperature on different germinative attributes of five *N. glauca* provenances representative of their natural distribution. The seeds were treated at a constant temperature (i.e., 18 °C, 22 °C, 26 °C, or 30 °C) in the absence of light for 40 days. The results show that in all the provenances, the germination ratio and energy increase linearly with temperature until reaching an optimum temperature (i.e., 22 °C), above which they decrease severely. At 22 °C, the response of average germination speed and germination vigor was significantly higher than with the other temperatures (performance of germination start day was not clear). The base temperature was around 18 °C and the maximum, above 30 °C, which may be close to thermo-inhibition. Given the threat of climate change, it is necessary to increase research in terms of the possible adaptation of this species to increased temperatures and prolonged periods of drought

## 1. Introduction

*Nothofagus glauca* (Phil.) Krasser (common name, hualo or roble maulino) is an endemic species of Central Chile that belongs to the Nothofagaceae family and is the most representative of the Mediterranean forests of its genus in South America. It is a deciduous, monoecious tree that can reach up to 30 m in height and 2 m in diameter [1], although at present it is difficult to find individuals that are more than 40 cm in diameter. The species is listed as vulnerable, and its populations are currently severely fragmented and trending toward decreasing [2].

The *N. glauca* forests have a discontinuous distribution in a latitudinal range of about 400 km, from 33°58′ S, 71°05′ W to 37°27′ S, 71°58′ W, although they are mostly concentrated in the Maule Region [3]. They are a transitional system between xerophytic formations and the southernmost temperate forests. This type of deciduous forest has adapted to the prolonged dry periods of summer and plays very important roles in the conservation of water and organic soil and in the biogeochemical carbon cycle as well as offering a great variety of ecological niches and habitats to the flora, fauna, and associated microbiota [4]. The natural range of this species has been considered a hotspot of biodiversity for conservation and is characterized by a great diversity of endemic species, although this has decreased to critical levels in terms of dominance and variability mainly because of anthropogenic factors [5]. The highest population density in Chile is concentrated in *N. glauca* distribution area, with the consequent pressure on natural resources, including the *N. glauca* forests.

The anthropogenic pressure has strongly shaped the landscape in the natural distribution area of *N. glauca* in recent years, affecting its spatial distribution, among other variables. Forty-five years ago, Urzúa [6] reported that there were 900,000 ha of these forests, whereas today, there are only 157,000 ha [3]. Another threat looming over these and other forests in the region is global climate change. Indeed, an increase in both temperatures and prolonged periods of drought has been recorded, factors that predispose the vegetation to being more prone to damage caused by biotic and abiotic agents. As an example, it can be noted that in the summer of 2017, 184,000 ha of the forest were consumed in a single fire, which affected an important part of this forest system [7]. In addition, considering that its regeneration by natural seeding is currently almost nonexistent, it is necessary to study its propagation by seeds to provide the necessary background to produce plants intended for afforestation and/or restoration of its populations. It is also important to consider the temperatures that limit the germination of seeds, especially if climate change is a factor that is affecting ecosystems.

Temperature is a crucial factor in the germination process of seeds [8]. It plays an important role in determining the periodicity of seed germination and the distribution of species (among other factors, it affects enzymatic activity) [9]. The germination rate generally increases linearly with temperatures up to an optimum temperature; subsequently, germination decreases severely with higher temperatures. Moreover, in the seed germination process, there are three levels of temperature: minimum, optimal, and maximum. The minimum, or base, temperature is the lowest temperature at which the seeds can germinate. The optimum temperature is the temperature at which the seeds reach their highest germination rate, and the maximum, or ceiling, temperature is the temperature above which the seeds cannot germinate [10]. The germination rate will increase between the minimum and optimum temperatures, whereas temperatures ranging from optimum to maximum will lead to a decrease in this attribute [11]. If the temperature is changing because of global climate change, it is evident that some species will have to adapt to these new environmental conditions, among other aspects, in the germination process.

*N. glauca* is a species whose seed production cycles are becoming longer, with years without seed production (unpublished data) and has seeds that present endogenous dormancy. There are mechanisms to break this condition, for example, through variable stratification periods at 4 °C (i.e., between 4 and 6 weeks) or by soaking them in gibberellic acid in concentrations from 0.1 to 0.8 g L^−1^ [12], reaching a germination ratio even above 95% using only viable seeds. However, the germination percentage may be different depending on the origin of the seeds [13]. Until today, no report has been made on the effect of temperature on the germination of any population of *N. glauca*. Therefore, this study aimed to analyze the effect of temperature in the germination process of seeds from five coastal provenances of *N. glauca*.

## 2. Results

**Figure 1:**
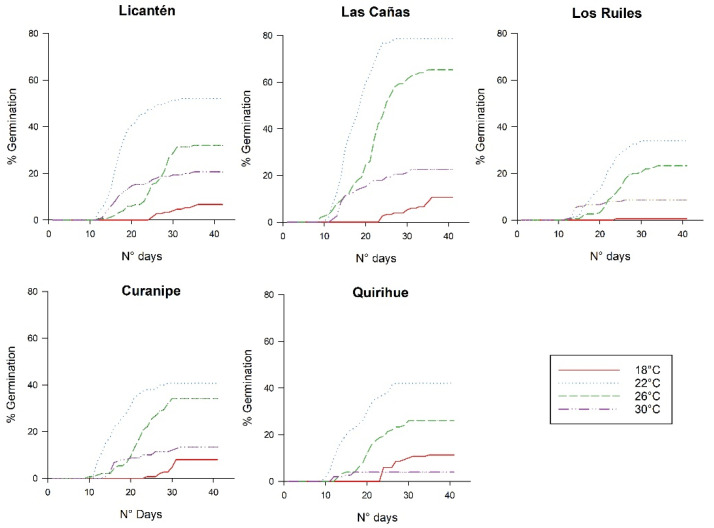
Cumulative germination percentage during 40 days for five *Nothofagus glauca* provenances treated at different temperatures in the absence of light.

**Figure 2:**
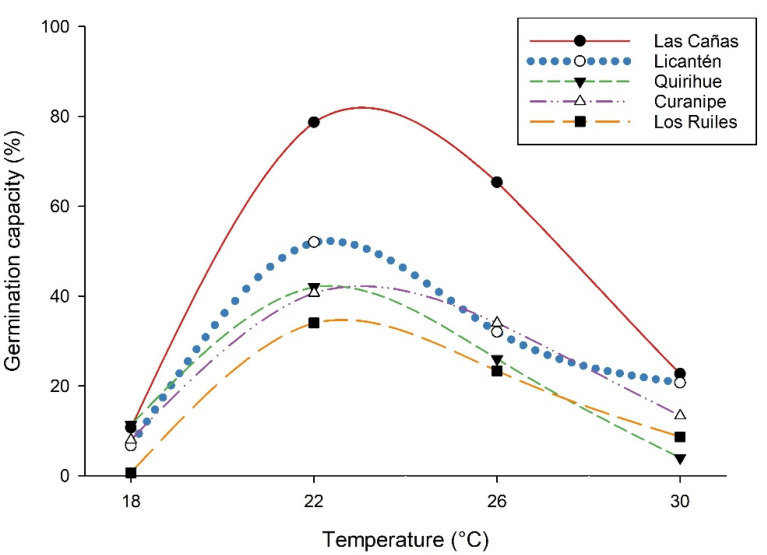
Final germination percentage of five provenances of *Nothofagus glauca* seeds treated at different temperatures in the absence of light.

The results show that temperature has a significant effect on the germination of *N. glauca* seeds from different provenances (Table 1, Figure 1 and Figure 2). In all the provenances, the highest germination percentage was obtained at 22 °C, although very different values were recorded in the germination ratio, from 34.0 ± 0.6% in the Los Ruiles provenance to 78 ± 0.3% in the Las Cañas provenance, observing the same tendency in the germinative energy. In general, in the northernmost provenances, a higher germination ratio was observed. These results show that *N. glauca* has a significant germination potential at 22 °C. In addition, the highest slope in the germination curves was observed at this temperature (Figure 2), which is an indicator that with endogenous application of gibberellic acid at 22 °C, latency is better overcome; it could be considered, then, that the optimum germination temperature for most provenances of *N. glauca* is around 22 °C (in the Las Cañas provenance, there were no significant differences between 22 °C and 26 °C).

In most of the provenances, once the seeds had begun to germinate, higher temperatures stimulated germination to an optimum temperature (22 °C), after which at 26 °C and 30 °C, the germination rate decreased. A clear pattern was also observed on the germination start day because of temperature, although seeds treated at 18 °C generally took longer to germinate. In general, average germination speed and vigor were higher at 22 °C, to later decrease with increasing temperatures; at 18 °C the lowest values were recorded in these two variables, although in some provenances, no significant differences were observed regarding those seeds treated at the highest temperature (30 °C).

## 3. Discussion

Seed germination is a process in which endogenous and exogenous factors intervene. On the one hand, two growth regulators that play a fundamental role in this process are gibberellic acid, a promoter of germination, and abscisic acid (ABA), which is an inhibitor. On the other hand, in addition to humidity and oxygen availability, temperature is the environmental factor with the greatest effect on the germination process of seeds, directly affecting their metabolism and germination speed [8]. With super-optimal temperatures, seed germination is delayed because a high level of ABA is maintained in the embryo and endosperm and the synthesis of gibberellic acid is suppressed [14,15]. The results of our research show a significant drop in germination at 30 °C, and it is likely that above this temperature, the seeds of *N. glauca* approach the thermo-inhibition process and prevent gibberellin biosynthesis.

The cardinal temperatures for germination are related to the environmental range of adaptation of a particular species and serve to match the germination time with the favorable conditions for the growth and subsequent development of the seedlings [10]. In our research in all the provenances studied, it was observed that from 18 °C, as the temperature increased, so did the germination until reaching a maximum at 22 °C, and then decreased. Thus, the optimum germination temperature for *N. glauca* would be at 22 °C, whereas the minimum, or basal, temperature would be around 18 °C, and the maximum, or ceiling, temperature would be above 30 °C. While the optimum temperature germination rate may vary between seed lots of the same species as a result of different genetic and environmental conditions [9], in the five provenances of *N. glauca*, this was not the case; the optimum temperature was clearly 22 °C, although for the Las Cañas provenance, it would be between 22 °C and 26 °C. It is striking that in the Los Ruiles provenance (area protected by the state), germination at 18 °C was practically null and the basal temperature for that provenance would likely be above that level. In the case of the southernmost provenance (Quirihue), at 30 °C, germination fell to levels below those reached at 18 °C; in this case, it would likely be closer to thermo-inhibition at that temperature. According to these results, it is probable that there is a genetic effect that conditions the germination process of the seeds.

Temperature not only plays a fundamental role in capacity and energy germination, but it also affects the germination start day, average germination speed, and germination vigor (Table 2). Once the seed has been rehydrated after imbibition, under suitable temperature conditions, physiological processes are triggered that allow the germination process to develop [8]. Although germination occurred at 18 °C, with variable rates according to the geographic origin of the seeds, the process is significantly slower at that temperature, taking at least 24 days to begin germination; instead, with the optimum temperature (i.e., 22 °C), it begins in some cases at 12 days. This same trend is also observed in both the average germination speed and vigor, meaning that the germination process of *N. glauca* is strongly conditioned by temperature. The germination ratio at the optimal temperature (i.e., 22 °C), is in the range found by other authors for the same species [12,13].

In the northernmost provenances, a higher percentage of germination was recorded, although no relationship was observed with the weight and morphometric characteristics of the seeds. In other species of the genus *Nothofagus*, it has been observed that the larger and heavier seeds have a greater germination ratio than those that are smaller and lighter, observing a clinal variation, although in a broader latitudinal distribution than that of this study [16]. This pattern was not observed in our study.

All species have a temperature range in which the germination process occurs. It has been described that for *N. glauca,* this range is likely between 10 °C and 30 °C [17]. On the one hand, in our research, we observed that the minimum temperature would be around 18 °C, especially for the Los Ruiles provenance, which is why it would be advisable to investigate the behavior of different provenances of this species under 18 °C. On the other hand, at 30 °C, germination strongly decreases, and above that level would be the maximum temperature and occurrence of thermo-inhibition. Consequently, temperatures under 18 °C and above 30 °C in the germination process of *N. glauca* should be evaluated.

Temperature is one of the main environmental factors that regulate seed physiology across plant taxa [10]. Because of climate change (i.e., increased temperatures and prolonged periods of drought), plants in general are being subjected to greater water stress, and therefore, their physiological processes are being affected. In the Mediterranean region of Chile, where *N. glauca* is distributed, there has been a significant increase in extremely hot events affecting the average temperature, and a deficit in rainfall [18]. *N. glauca* is widely known for its strong tendency for alternate bearing, which severely affects the fruit yield from year to year, and it has been observed that the cycles in seed production are becoming longer, with years without seed production (unpublished data). Given the threat of climate change on the reproductive cycle of the species and considering the effect that temperature has on the germination of *N. glauca* seeds, it is urgent and necessary to study in greater depth the adaptation capacity that this species would have to these new environmental conditions. Santelices, Espinoza, Magni, Cabrera, Donoso, and Peña [13] reported that the intra-provenance variability of *N. glauca* is systematically greater than that of inter-provenance, indicating a high potential capacity of the species to adapt to climate change. However, these authors affirm that there are differences in germination between Andean and coastal origins (in our research, we evaluated only costal provenances); this reaffirms the need to deepen research on the potential adaptation of *N. glauca* to climate change and the capacity of the species to regenerate and self-perpetuate.

## 4. Material and Methods

### 4.1. Seed Collection and Preparation

**Figure 3:**
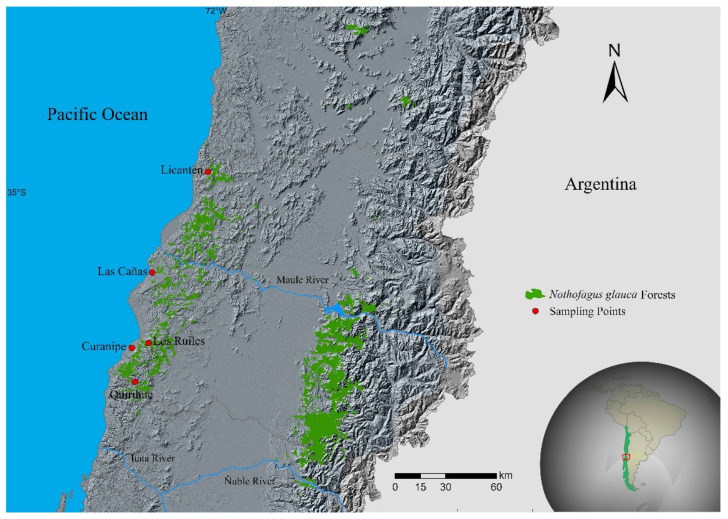
Seed provenances of *Nothofagus glauca*.

In March 2017, mature seeds were collected from different provenances of *N. glauca* in the Maule Region of Chile (Table 3, Figure 3), except those from Licantén, which were collected in March 2015. Seeds were transported to the laboratory, where they were manually separated from the rest of the plant material and the damaged seeds were discarded; then, they were weighed, dried, and stored in the dark in glass containers in an environment at 4 °C until they were used (i.e., January 2020). The standards of the International Seed Testing Association (ISTA) were followed to characterize the seeds [19]. One hundred seeds were weighed separately for eight repetitions to determine the weight of the seeds, which was expressed as the average weight of 1000 seeds. Then, the average weight of 1000 seeds and its equivalence in number of seeds per kilogram were calculated. In addition, the dimerous seeds were measured for length and width, and the trimerous seeds for length, width, and thickness (Table 2). To break the dormancy, the seeds were soaked in a 200 mg L^−1^ gibberellic acid solution (Giberplus^®^ Tabletas, Anasac Chile S.A., Santiago, Chile) for 24 h before starting the germination tests [12].

The mean annual precipitation and temperature were obtained from WorldClim (version 2) at a spatial resolution of 30 s (~1 km^2^) by interpolation of the records of meteorological stations from 1970 to 2000 [20].

### 4.2. Germination Experiments

The study was carried out in a laboratory at the Universidad Católica del Maule, Talca, Chile (35°26′10″ S, 71°37′13″ W, 131 m a.s.l.), during January and February 2020. The seeds were soaked in a Gibberellic Acid (GA_3_) solution at 200 mg L^−1^ for 24 h using distilled water, and those that floated were excluded as they were considered unviable. To determine the effect of temperature on the germination of *N. glauca* seeds from different provenances, four different temperatures were tested: 18 °C, 22 °C, 26 °C, and 30 °C. The cultivation was carried out in germinating chambers in the absence of light, maintaining fixed temperatures according to each treatment and using filter paper as the substrate. To not interfere with the treatments, the ambient temperature of the laboratory was constantly maintained below 16 °C. Irrigation was manual, and care was taken that the seeds were always wet.

The germination process was monitored daily until germination ceased over a period of 40 days. Seeds were considered to have germinated when the emerging radicles were over 2 mm long. The following germination parameters, adapted from [21,22], were calculated:

(1) $$GR=\left[\frac{Sg}{Ss}\right]\times 100$$

Germination ratio (GR) (1): represents the final percentage of seeds that germinate (Sg) in relation to the total number of seeds sown (Ss):

Germination energy (GE): accumulated percentage of germination on the day when the maximum value occurs (maximum value is maximum ratio from cultivated germination percentage on day X divided by X).

Germination start day (GSD): the time elapsed from the sowing of the seeds to the germination of 5% of the sown seeds.

Average germination speed (AGS) [2]: corresponds to the average number of germinated seeds per day, calculated by the expression:

(2) $$AGS=\sum_{1}^{k}\frac{ni}{ti}$$

where *ni* corresponds to the number of seeds germinated in the *i*th data collection, *ti* is the time (in days) of the *i*th data collection, and *k* is the time (in days) of the germination test duration.

Germination vigor (GV): reflects in a single value the changes in the germination peak, the total germination, and the germination speed, calculated as the product between the maximum value and the average germination speed.

### 4.3. Trial Design and Statistical Analysis

There were two factors tested in the trial: temperature (four levels) and provenance (five levels). The factorial combination of 20 treatments (4 × 5) was replicated three times in a split-plot design. Fixed effects were randomly assigned within subplots (considering the homogeneity of the temperature, whole plots were not randomized). There were 50 viable seeds per factorial combination, giving a total of 150 seeds per treatment. Temperature was applied to the whole plots and provenance, to the subplots. The treatments imposed follow:

- Temperature: 18 °C, 22 °C, 26 °C, and 30 °C
- Provenance: Licantén, Las Cañas, Los Ruiles, Curanipe, and Quirihue

Analyses of variance (ANOVAs) and comparisons of the means were conducted using the general linear model (GLM) procedure from the statistical software SPSS for Windows (SPSS, Chicago, IL, USA). Mean values with significant differences were compared using the Tukey test at the 5% significance level.

## 5. Conclusions

Based on the results of this research, it can be concluded that temperature is an abiotic factor that significantly affects the germination of *N. glauca*. In the absence of light conditions, the optimum germination temperature for all the provenances studied was 22 °C. By maintaining the temperature at 18 °C, germination was induced, although at a low percentage. At 30 °C, the germination percentage was also low, and above this level is the maximum germination temperature and risk of thermo-inhibition.

## Figures and Tables

**Table 1:** Effect of temperature on different germination parameters of *Nothofagus glauca* (mean ± SE). Different letters indicate significant differences by Tukey’s multiple comparison test (*p* < 0.05).

Provenance	Temperature (°C)	Germination Ratio (%)	Germination Energy (%)	Germination Start Day	Average Germination Speed (Seed/Day)	Germination Vigor
Licantén	18	6.7 ± 0.67 d	6.8 ± 0.33 d	25.0 ± 0.0 c	0.1 ± 0.22 c	0.0 ± 0.0 d
	22	52.0 ± 0.01 a	45.0 ± 2.26 a	11.0 ± 0.94 a	1.5 ± 0.07 a	2.4 ± 0.11 a
	26	32.0 ± 0.01 b	31.4 ± 1.44 b	16.0 ± 0.94 b	0.7 ± 0.03 b	0.9 ± 0.04 b
	30	20.7 ± 0.67 c	14.6 ± 0.71 c	13.0 ± 0.47 ab	0.6 ± 0.03 b	0.4 ± 0.02 c
Las Cañas	18	10.7 ± 0.54 c	6.2 ± 0.11 c	24.3 ± 0.27 b	0.2 ± 0.01 d	0.1 ± 0.0 d
	22	78.7 ± 0.54 a	77.1 ± 3.42 a	12.0 ± 0.94 a	2.4 ± 0.11 a	9.1 ± 0.41 a
	26	65.3 ± 0.54 a	57.5 ± 2.97 a	10.7 ± 0.72 a	1.7 ± 0.08 b	4.0 ± 0.2 b
	30	22.7 ± 0.54 b	14.0 ± 0.65 b	12.0 ± 0.47 a	0.7 ± 0.04 c	0.6 ± 0.03 c
Los Ruiles	18	0.7 ± 0.54 d	0.7 ± 0.54 d	0.0 ± 0.0 c	0.0 ± 0.0 d	0.0 ± 0.0 d
	22	34.0 ± 0.0 a	31.2 ± 0.0 a	16.0 ± 0.94 ab	0.8 ± 0.04 a	1.3 ± 0.05 a
	26	23.3 ± 0.54 b	19.3 ± 0.9 b	21.0 ± 0.94 b	0.5 ± 0.02 b	0.5 ± 0.03 b
	30	8.7 ± 0.54 c	6.7 ± 0.49 c	14.0 ± 0.47 a	0.3 ± 0.01 c	0.2 ± 0.02 c
Curanipe	18	8.0 ± 0.94 d	8.0 ± 0.42 b	24.3 ± 1.19 b	0.1 ± 0.01 d	0.0 ± 0.0 d
	22	40.7 ± 0.54 a	34.6 ± 1.62 a	12.0 ± 0.47 a	1.3 ± 0.06 a	2.3 ± 0.11 a
	26	34.0 ± 0.0 b	34.0 ± 0.0 a	13.0 ± 0.47 a	0.8 ± 0.03 b	1.3 ± 0.06 b
	30	13.3 ± 0.54 c	8.1 ± 0.38 b	14.0 ± 0.47 a	0.4 ± 0.02 c	0.2 ± 0.01 c
Quirihue	18	11.3 ± 0.54 c	10.8 ± 0.47 c	24.0 ± 0.94 b	0.2 ± 0.02 c	0.3 ± 0.01 c
	22	42.0 ± 0.0 a	35.4 ± 1.72 a	10.0 ± 0.47 a	1.3 ± 0.08 a	5.2 ± 0.25 a
	26	26.0 ± 0.0 b	23.3 ± 1.1 b	13.0 ± 0.47 a	0.6 ± 0.03 b	1.7 ± 0.08 b
	30	4.0 ± 0.0 d	3.8 ± 0.14 d	12.0 ± 0.47 a	0.1 ± 0.01 d	0.1 ± 0.01 d

**Table 2:** Geographic and climatic data for provenances sampled.

Provenance	Latitude	Longitude	Elevation (m a.s.l.)	M.A.T ^1^ (°C)	M.A.R ^2^ (mm Year^−1^)
Licantén	34°55′49″ S	72° 5′11″ W	403	12.5	829
Las Cañas	35°27′27″ S	72°28′11″ W	165	12.9	831
Los Ruiles	35°50′2″ S	72°30′36″ W	202	12.4	851
Curanipe	35°51′24″ S	72°37′13″ W	129	13.1	817
Quirihue	36°2′18″ S	72°36′35″ W	342	12.0	898

^1^ M.A.T.: mean annual temperature; ^2^ M.A.R.: mean annual rainfall.

**Table 3:** Weight and morphometric characterization of the seeds from five provenances of *Nothofagus glauca* (mean ± Standard Error).

Provenance	Weight of 1000 Seeds (g)	Number of Seeds per Kilogram	Dimerous Seeds		Trimerous Seeds		
			Length (mm)	Width (mm)	Lenght (mm)	Width (mm)	Thickness (mm)
Licantén	468.3 ± 0.3	2136 ± 10.4	17.1 ± 1.8	14.2 ± 1.8	17.2 ± 0.1	10.4 ± 0.1	9.7 ± 0.1
Las Cañas	537.5 ± 0.4	1861 ± 14.0	19.5 ± 0.1	12.1 ± 0.2	19.0 ± 0.1	10.8 ± 0.1	9.9 ± 0.1
Los Ruiles	683.7 ± 0.5	1463 ± 10.3	19.7 ± 1.6	13.2 ± 1.8	19.6 ± 0.2	11.3 ± 0.2	11.2 ± 0.1
Curanipe	395.5 ± 0.3	2530 ± 9.9	19.5 ± 0.3	11.3 ± 0.2	17.5 ± 0.3	10.3 ± 0.1	8.2 ± 0.2
Quirihue	379.0 ± 0.4	2641 ± 14.9	19.3 ± 0.1	12.1 ± 0.1	18.5 ± 0.2	9.7 ± 0.1	9.1 ± 0.1

## Data Availability

No new data were created or analyzed in this study. Data sharing is not applicable to this article.
